# The follow-up of myocardial injury and left ventricular function after spontaneous coronary artery dissection

**DOI:** 10.3389/fcvm.2023.1276347

**Published:** 2023-11-14

**Authors:** Gordana Krljanac, Svetlana Apostolovic, Marija Polovina, Ruzica Maksimovic, Olga Nedeljkovic Arsenovic, Nemanja Djordjevic, Stefan Stankovic, Lidija Savic, Aleksandra Djokovic, Mihajlo Viduljevic, Sanja Stankovic, Milika Asanin

**Affiliations:** ^1^ Cardiology Clinic, University Clinical Center of Serbia, Belgrade, Serbia; ^2^ Faculty of Medicine, University of Belgrade, Belgrade, Serbia; ^3^ Coronary Care Unit, Cardiology Clinic, University Clinical Center of Nis, Nis, Serbia; ^4^ Faculty of Medicine, University of Nis, Nis, Serbia; ^5^ Center for Radiology and Magnetic Resonance Imaging, University Clinical Center of Serbia, Belgrade, Serbia; ^6^ Department of Cardiology, University Hospital Center Bezanijska kosa, Belgrade, Serbia; ^7^ Center for Medical Biochemistry, University Clinical Center of Serbia, Belgrade, Serbia; ^8^ Faculty of Medical Sciences, University of Kragujevac, Kragujevac, Serbia

**Keywords:** spontaneous coronary artery dissection, myocardial injury, left ventricular function, follow-up, echocardiography, cardiac magnetic resonance

## Abstract

Monitoring patients with spontaneous coronary dissection (SCAD) is critical in their care, as there are no accepted recommendations. To this end, finding clinical or imaging predictors of recurrent events in these patients is essential for predicting adverse events and guiding treatment decisions between conservative medical therapy and percutaneous coronary intervention. Myocardial injury and left ventricular function after SCAD can be variable parameters that require monitoring. Echocardiography and cardiac magnetic resonance are two useful imaging techniques to do so. This review aims to analyze previously published results on monitoring myocardial injury and left ventricular function in SCAD patients while highlighting the potential benefits of contemporary imaging techniques that could further improve patient care in the future.

## 1. Introduction

Following spontaneous coronary artery dissection (SCAD) during hospitalization and long-term follow-up is essential to identify clinical or imaging parameters as predictors of recurrent events. According to the research of Saw et al. in 2017 (1), the most common outcomes for SCAD patients were mortality, recurrent myocardial infarction, recurrent SCAD, stroke or transient ischemic attack, revascularization procedures such as angioplasty and stenting, ventricular arrhythmias, and sudden cardiac death (SCD).

In the European Society of Cardiology/Acute Cardiovascular Care Association/SCAD Study Group position paper from 2018 (2), Adlam et al. recommend that SCAD patients presenting with recurrent chest pain should be carefully assessed via serial electrocardiography (ECG), high-sensitivity troponin measurement, and coronary angiography imaging following the assessment of the physician (2). How follow-up coronary imaging should be utilized to guide subsequent management in determining SCAD healing remains uncertain (2). Therefore, assessing left ventricular (LV) systolic function is significant and mandatory to guide medical and potentially invasive or device therapy (2). Besides LV systolic function, infarct size strongly predicts the prognosis after SCAD.

In this review, we summarize published literature on the role of monitoring myocardial injury and LV function in SCAD patients and its significance for outcomes of patients while also highlighting potential benefits provided by contemporary imaging techniques that could further improve patient care.

Contemporary Medline papers published since 2010 were searched using Medical Subject Headings and keywords, focusing on SCAD, imaging methods, and outcomes. We also manually searched the reference lists of relevant studies and reviews to find relevant citations. The authors evaluated all citations to select the most appropriate ones to be included in this review.

## 2. Challenges in the diagnosis and following patients with SCAD

Echocardiography and cardiac magnetic resonance (CMR) imaging are two of the most useful diagnostic tools available for diagnosing and following up SCAD patients. These imaging methods provide invaluable information about the structure, function, and perfusion of the myocardium that is affected by SCAD-related ischemia. They can also help detect any underlying conditions contributing to SCAD development or progression. Their high-resolution images offer a comprehensive evaluation essential in identifying potential treatment strategies and monitoring response to therapy over time.

When diagnosing and monitoring SCAD, one may encounter challenges distinguishing it from Takotsubo cardiomyopathy (TC), as their features bear some resemblance. In 2017, Buccheri and Zambelli (3) identified several distinguishing features of SCAD and TC. These include a higher incidence in females, a correlation with stressful events, and clinical manifestations like angina or acute coronary syndromes. In addition, the absence of acute plaques on coronary angiography characterized both diseases. Non-obstructive coronary artery disease has been identified in TC. However, the appearance of SCAD on angiography can manifest in various forms, ranging from the classic hallmark of contrast dye staining with multiple radiolucent lumens to diffuse and smooth narrowing. This can cause varying degrees of vessel obstruction and focal stenosis, resembling an atherosclerotic plaque (3). Two main hypotheses explain the possible overlap between SCAD and TC. First, SCAD could be the stressful event that leads to TC, similar to myocardial infarction (4). The second hypothesis is that external torsion forces and mechanical solicitations associated with typical wall motion abnormalities in TC could cause the dissection of the intima, especially in segments located at the border between basal hyperkinesia and mid-apical akinesia (5). When diagnosing TC in the first few days of the disease, the most important characteristics are “LV apical ballooning” and “normal coronary arteries.” On the other side, Chou et al. (6) presented that patients with SCAD had post-ischemic myocardial stunning that extended beyond the supply region of the dissected coronary arteries. They also found that wall motion myocardial dysfunction and stunning had features consistent with SCAD (6). Shams and Henareh (7) explained that SCAD may be a missed diagnosis when overlapping with TC. The diagnosis of SCAD and TC is not mutually exclusive, and both conditions may rarely occur in the same patient.

Identifying short-term and long-term indicators of a negative outcome following treatment for SCAD patients is crucial. Studies have shown that the long-term mortality rate for those who survive SCAD is relatively low, ranging from 1.2% to 8% over 3.1–10 years (1, 8–12). However, the overall rate of major adverse cardiac events (MACE) in SCAD patients is significant, although it varies greatly across different published studies (19.9%–47.4%) over 3.1–10 years (1, 9–11).

After suffering from SCAD, the outpatient evaluation should be personalized based on the patient's clinical status, extent and location of dissection and myocardial injury, and symptoms. The American Heart Association's position paper published in 2020 (12) suggests that clinical outpatient visits could focus on important aspects of patients’ recent and past history, family history, and physical and diagnostic evaluation (12) (Table 1).

**Table 1:** Recommendation for outpatient evaluation of patients after SCAD [Modified from (12)].

**1. Characteristics of SCAD illnesses** • Clinical presentation • Management of illness • Description of possible precipitants of illness • Mental health status
**2. Medical history** • Prior myocardial infarction • Prior cardiovascular diseases • Presence of fibromuscular dysplasia or other arterial abnormalities • Migraine • Connective tissue disorder diagnoses or signs/symptoms • Rheumatologic/autoimmune disorders • Atherosclerotic risk factors
**3. Reproductive history** • Number of pregnancies and deliveries • Pregnancy/delivery complications (e.g., pre-eclampsia, hypertension in pregnancy, diabetes, and so on) • Infertility • Infertility treatment • Sex hormone use history
**4. Family history** • Premature myocardial infarction • Premature stroke • Sudden cardiac death • Arterial abnormalities (e.g., aneurysms, dissections, and fibromuscular dysplasia) • SCAD • Connective tissue disorders
**5. Socio-epidemiological data** • Physical activity • Alcohol and/or drug abuse

**Figure 1:**
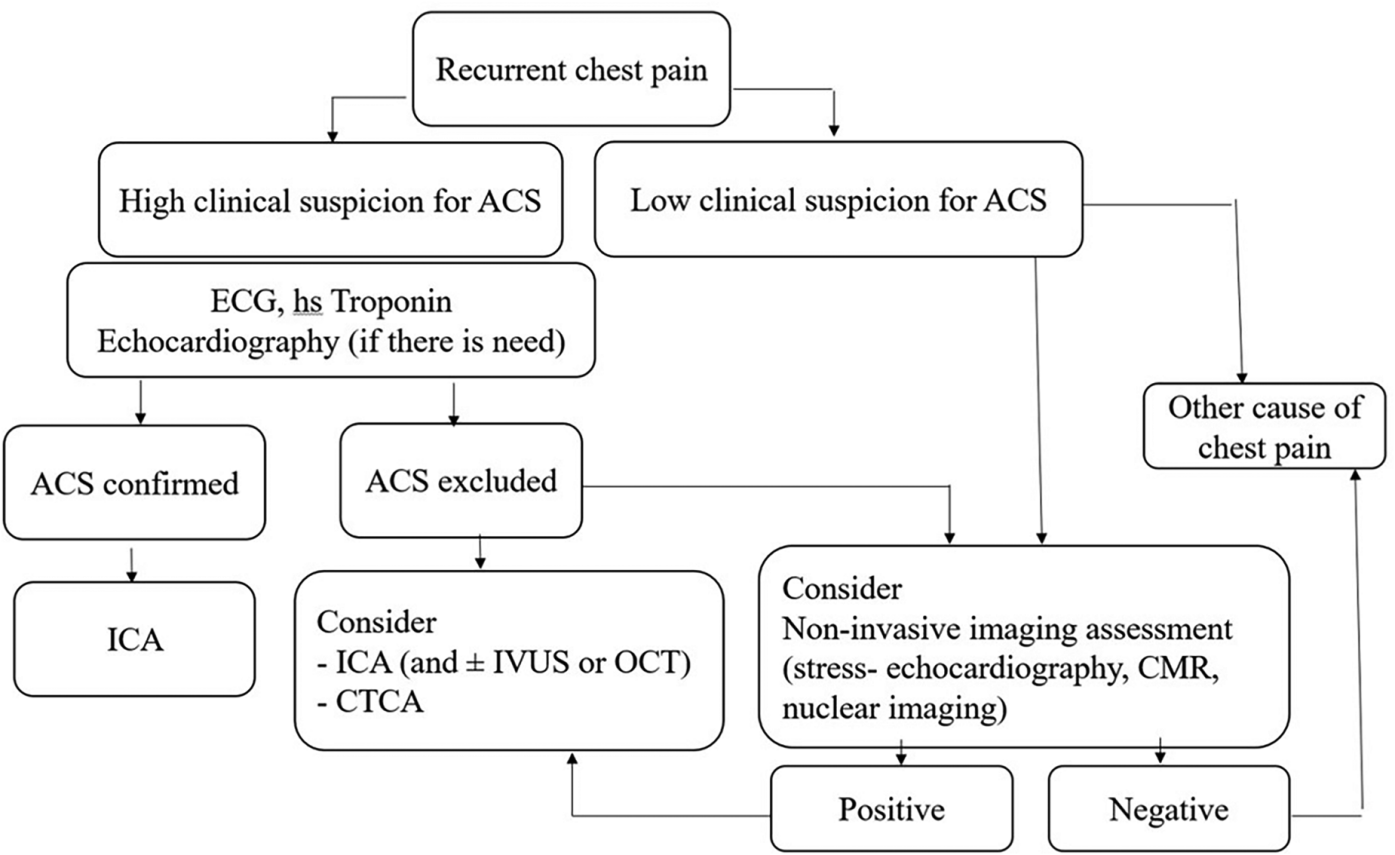
Proposed algorithm for evaluation of patients with recurrent chest pain following previous SCAD episode [Designed according to reference (12)]. ACS, acute coronary syndrome; CMR, cardiac magnetic resonance; CTCA, computer tomography coronary angiography; hs-troponin, high-sensitivity troponin; ICA, invasive coronary angiography; OCT, optical coherence tomography; IVUS, Intravascular ultrasound; SCAD, spontaneous coronary artery dissection.

Chest pain syndromes may occur during early or late follow-up after SCAD. This position paper released an algorithm to assess and treat repeated SCAD symptoms. The approach considers the type of symptoms and prioritizes accurate diagnosis and symptom relief while minimizing the risk of harm from medical iatrogenic intervention (Figure 1).

## 3. Diagnostic imaging methods used in SCAD patients

SCAD is a potentially serious condition where a tear occurs within the layers of the coronary artery, leading to reduced blood flow and possible myocardial damage.

The commonly used imaging methods in SCAD could be classified into two groups.

The first group of imaging methods for assessing coronary anatomy, identifying the type and severity of SCAD, and detecting perfusion abnormalities of the coronary artery include the following:

1. Invasive coronary angiography is used to visualize coronary arteries and detect abnormalities, including dissections.
2. Computed tomography angiography (CTA) is a non-invasive imaging method that uses x-rays to create detailed cross-sectional images of the coronary arteries. It can be used to visualize the extent of coronary artery dissection and assess blood flow.
3. Intravascular ultrasound (IVUS) uses a tiny ultrasound probe attached to a catheter threaded into the coronary arteries to visualize coronary arteries.
4. Optical coherence tomography (OCT) is an imaging technique that uses light waves to create detailed cross-sectional images of the coronary arteries.

The first imaging group is focused on SCAD diagnostic challenges and increases awareness of the various SCAD angiographic appearances from Types 1 to 4 (12, 13). Type 1 is the most common presentation of a longitudinal filling defect due to the intimal flap. Type 2 is characterized by diffuse long smooth tubular stenosis caused by intramural hematoma without apparent dissection. Type 3 presents as multiple tubular lesions caused by intramural hematoma that can mimic atherosclerosis and require intravascular imaging to diagnose. Type 4 has been described as a complete vessel occlusion (13).

The second group of imaging tools used to assess the infarct size, myocardial perfusion, and LV function in SCAD comprises the following:

1. CMR is a non-invasive imaging method that can provide detailed information about the myocardial structure, function, and blood flow. It can help identify areas of ischemia and assess myocardial viability.
2. Single-photon emission computed tomography (SPECT) or positron emission tomography (PET) assesses blood flow to the myocardium and identifies areas of reduced perfusion due to SCAD.
3. Echocardiography is a non-invasive imaging method that uses sound waves to create real-time images of the structure and function. It can help assess heart wall motion abnormalities and identify the presence of any fluid collection around the heart (pericardial effusion).
4. Strain or myocardial deformation is a comprehensive technic for assessing global and regional myocardial function in ischemic myocardium and SCAD.

According to Hayes et al. (14), the recommended diagnostic tools for assessing the infarct size and left ventricle (LV) function after SCAD are echocardiography and CMR (14). Defining the parameters that could be predictors of the prognosis of SCAD patients is of crucial importance.

When analyzing patients with SCAD, it is important to consider coronary microvascular dysfunction (CMD). This term covers various conditions, such as non-coronary reflux, microvascular obstruction (MVO), intramyocardial hemorrhage (IMH), and microvascular injury (MVI). Even after prompt epicardial recanalization of the infarct-related artery, CMD can still occur and increase the risk of cardiovascular events regardless of the epicardial disease status. To determine functional recovery after acute myocardial infarction (AMI), Nijveldt et al. (15) identified early MVO on first-pass perfusion imaging and late MVO on late gadolinium-enhanced imaging determined by CMR as important prognostic parameters.

MVO and IMH were found in the same area as SCAD involvement during angiography (16). Although larger studies are needed to confirm this, the emergence and withdrawal mechanisms of MVO and IMH in SCAD-related myocardial infarction with ST-elevation (STEMI) may differ in comparison to type 1 STEMI patients. The exact pathophysiological mechanism of post-ischemic CMD is still being debated (17, 18), along with future opportunities for preventing or treating CMD in STEMI patients. Pathophysiological mechanisms of CMD in AMI are divided into intraluminal obstruction (such as thromboembolism, plugging, and vasospasm) and extravascular expression (such as IMH, interstitial and cellular edema, and increased end-diastole pressure of the left ventricle) (18). Future research may focus on identifying the predominant pathophysiological mechanisms that contribute to CMD in SCAD.

## 4. Myocardial infarct size in SCAD patients

The formation of myocardial infarct size in patients with SCAD could be a balanced process and often unpredictable in accordance with the clinical course and outcome.

Due to the unique nature of SCAD and its challenges in diagnosis and management, guidelines for the usage of imaging methods to follow the infarct size may not be clearly defined.

The first present small study about CMR revealed that it can be useful in confirming the diagnosis of myocardial infarction in cases where there is uncertainty (19). Moreover, CMR can also evaluate the degree of myocardial involvement and assess for any other accompanying causes and outcomes (19).

**Figure 2:**
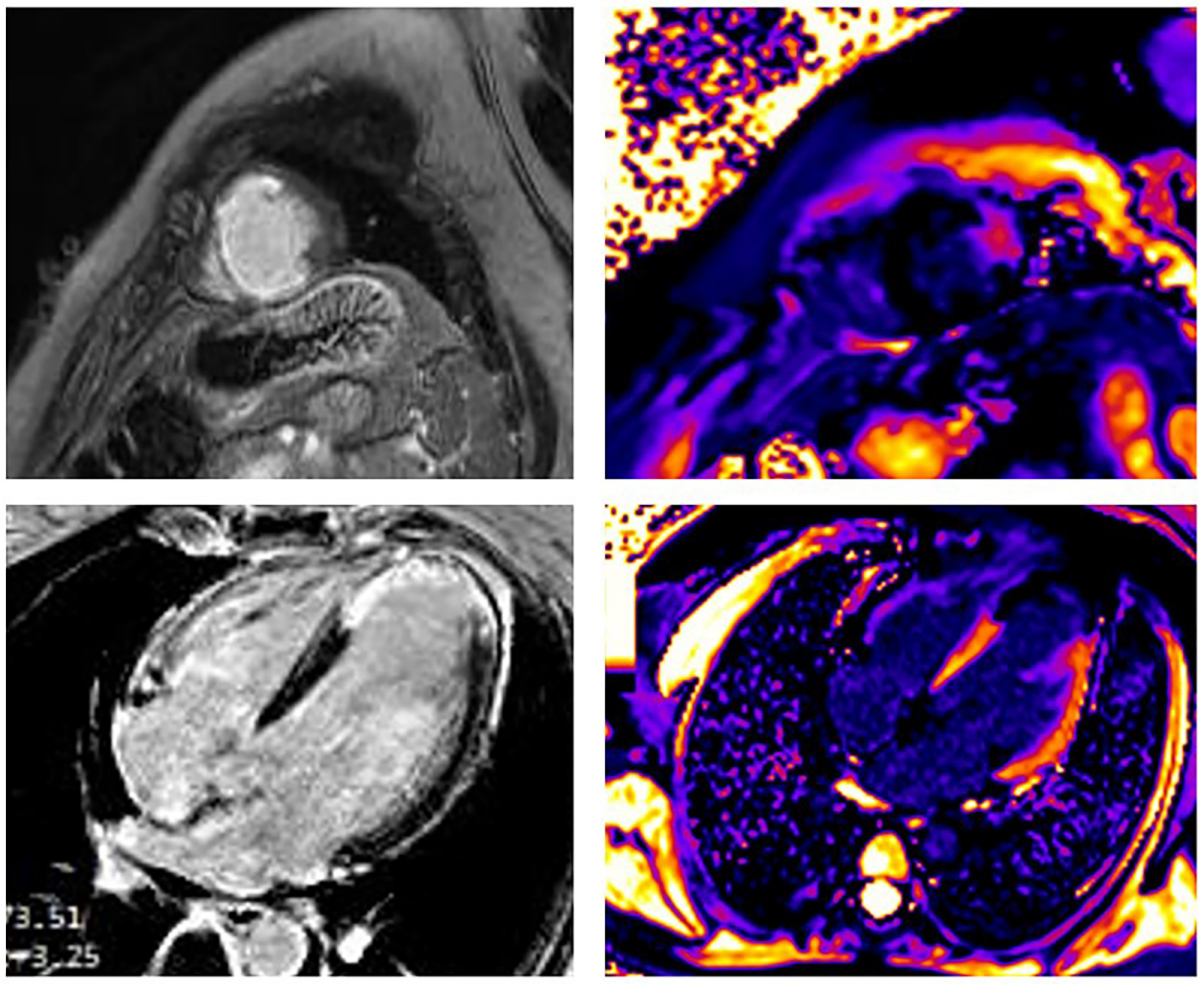
A 42-year-old woman presented as STEMI anterior localization SCAD on left anterior artery type 4 and TIMI flow 0, treated with percutaneous transluminal coronary angioplasty (PTCA) without implantation of stents. Late gadolinium enhancement (LGE) was seen in the sub-endocardium in the medio-apical part of the septum apical parts of inferior and posterior walls and transmural in the apical part of the septum and anterolateral walls of LV. Post-contrast T1 mapping identified the zone of fibrosis, which was the zone of infarction and was also present in apical segments of LV. In addition, the peri-infarct area had a high T1 signal. The size of fibrosis (infarct size) was 13%.

**Figure 3:**
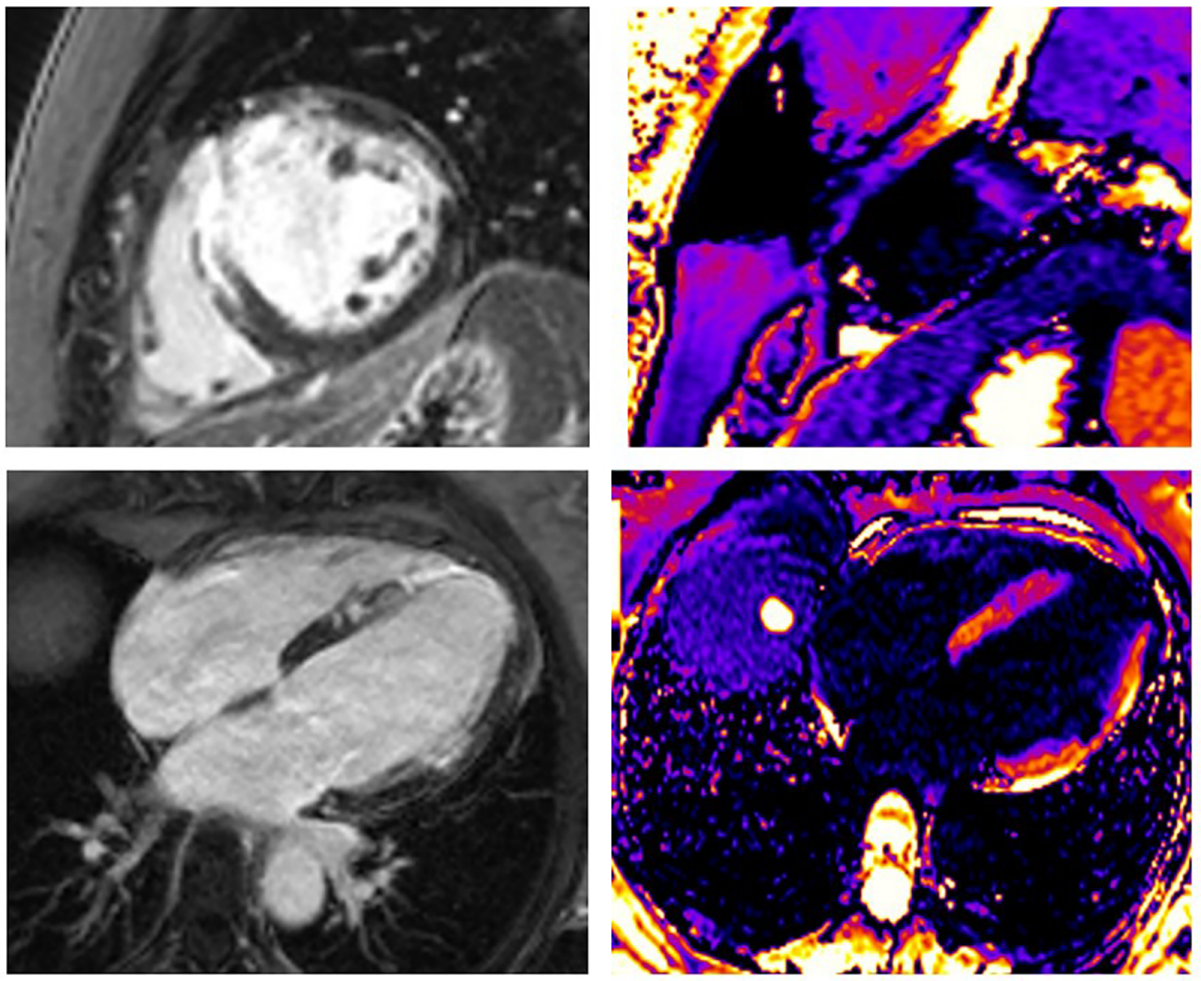
A 43-year-old woman presented as STEMI anterior localization, SCAD on left anterior artery type 3, and TIMI flow 0, treated with percutaneous coronary intervention (PCI) and implanted four stents guided with intravascular ultrasound (IVUS). LGE was seen in the medio-apical part of the septum, apical parts of the anterior wall transmurally, and in the medial parts of the anterior wall in the sub-endocardial layer. Using post-contrast T1 mapping, the zone of fibrosis, which was the zone of infarction, was also present in the same segments. The size of fibrosis (infarct size) was 17%.

**Figure 4:**
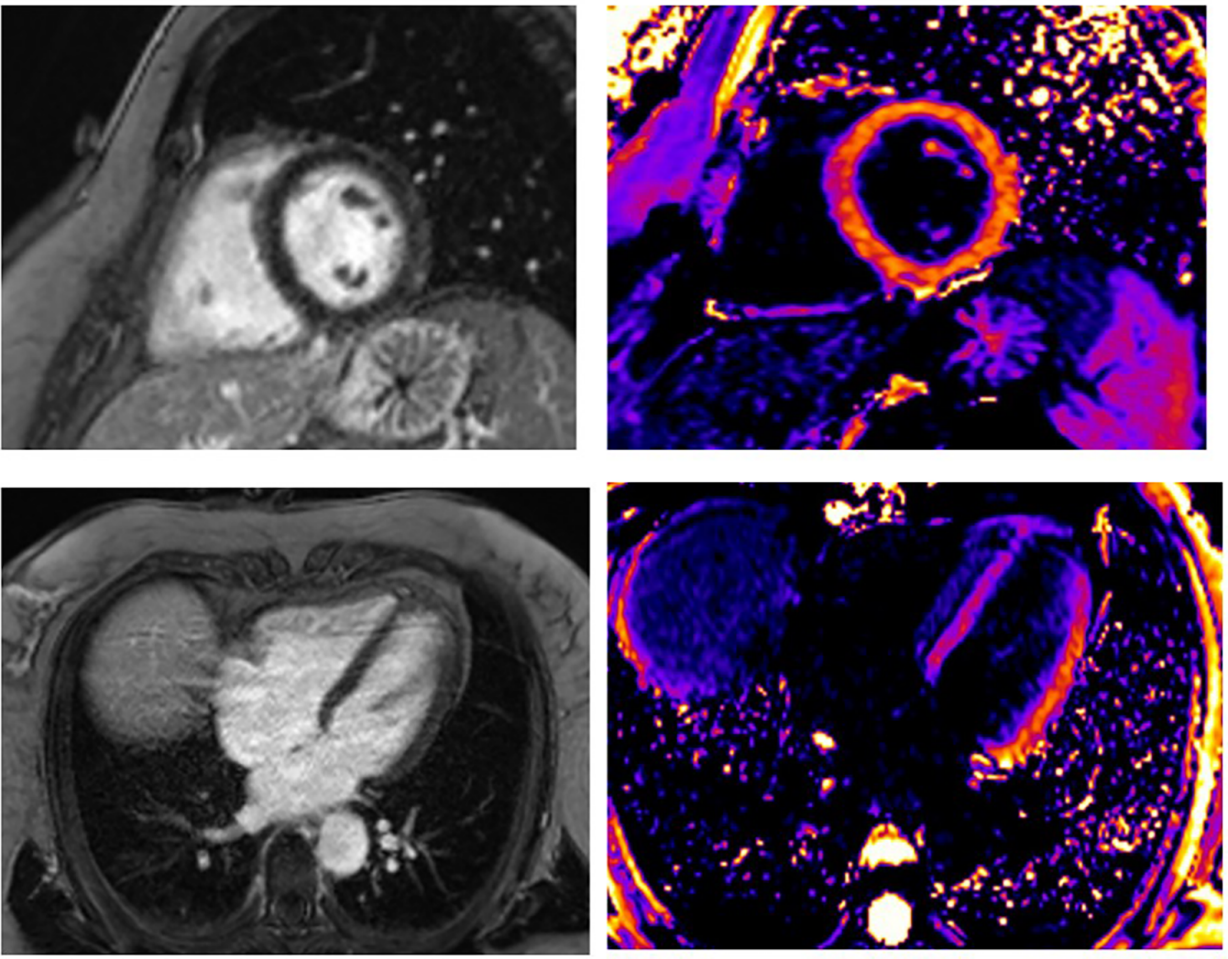
A 55-year-old woman presented STEMI anterior localization, SCAD on left anterior artery type 2A, and TIMI flow 2, treated with conservative medical therapy. There was no LGE in the myocardium of LV. Using post-contrast T1 mapping, the values were normal. The infarct size was 0%.

The occurrence of myocardial injury in SCAD, which presents as myocardial infarction, may not always be visible and depends on the type of SCAD. It is in accordance with thrombolysis in myocardial infarction (TIMI) flow and the speed of healing coronary disease. Figures 2–4 present three patients with different types of SCAD and myocardial infarct sizes, which depend on that.

Infarct size is usually quantified semi-automatically on late gadolinium enhancement (LGE) imaging using the full-width half-maximum technique such that infarct size = (LGE mass/total LV mass) × 100. Al-Hussaini et al. (20) defined the infarct size using CMR as a small or large infarct in accordance with the percentage of LV mass (>10% or ≤10%) (20). Approximately 39% of SCAD patients have no detectable LGE, and univariate predictors of infarct size are TIMI 0/1 vs. TIMI 2/3 flow, STEMI vs. non-ST-segment elevation myocardial infarction (NSTEMI) presentation, and patients with a Beighton score ≥4 (20). Similarly, multivariable predictors of any myocardial infarction were STEMI presentation, Beighton score ≥4, and multivessel involvement (20). The Beighton score is a clinical score used to assess hypermobility and joint laxity and is measured on a scale of 1–9 (21).

The results of multivariate modeling show that certain factors are associated with larger myocardial infarction size (>10% of LV mass present on LGE), specifically in cases of STEMI presentation, initial TIMI 0/1 flow, multivessel SCAD, a Beighton score of at least 4, and multivessel involvement (21). According to Al-Hussaini et al. (20), SCAD infarcts are generally small, and only 6.4% of SCAD patients experience infarct sizes greater than 20% of the LV mass. Interestingly, when comparing female SCAD patients presenting with STEMI and female Type 1 STEMI patients under 75 years of age, similar-sized myocardial infarcts were found in both groups (20).

The myocardial infarct size can be changeable in SCAD survivors and STEMI survivors, depending on the lumen size and coronary flow through the artery. In 2019, Jackson et al. (22) showed a mechanism of false lumen (FL) formation in SCAD, which could be fenestrated and non-fenestrated, and SCAD STEMI with less thrombosed true lumen than type 1 STEMI. This may explain why SCAD STEMI patients tend to have smaller infarcts and less LGE endocardial involvement than STEMI patients with a typical pattern of endocardial involvement (20). In another study, Al-Hussaini et al. (20) explained that the size of a chronic infarct in SCAD patients can vary depending on the conditioning effect and collateralization caused by a previously fixed stenosis, as in Type 1 STEMI. Treatment delays and the absence of conditioning effect and collateralization may cause an increased infarct size in SCAD STEMI patients, especially those with poor TIMI flow, making them more susceptible to larger infarcts similar to those in Type 1 STEMI patients (20). However, there may be exceptions among patients.

Sustained inflammation after myocardial infarction leads to LV remodeling and progressive cardiac dysfunction, such as heart failure. The extended and inappropriate immune response in SCAD, as well as in STEMI, could be the explanation for the future LV remodeling and formation of the infarct scar size (22). Increased proinflammatory biomarkers such as tumor necrosis factor (TNF)-α, interleukin (IL)-1β, IL-6, and monocyte chemoattractant protein-1 are strongly related to inappropriate healing of the myocardium after myocardial infarction and progressive cardiac dysfunction (23). In previous experimental studies, the authors found two patterns of immune response. The first pattern consisted of the rapid CD4+ T-cell response with maximal levels seen at 3 days post-MI, which returned to baseline levels by 14 days (24). However, in the case of developing heart failure, the second phase of CD4+ T-cell activation occurred, leading to further damage of the cardiomyocytes (25). The acknowledgment of beneficial and harmful contributions of specific immune cells and cytokines on myocardial infarction size leads to novel therapeutic targets that can modulate the immune system (26). Mechanisms that can boost myocardial recovery and repair are important in cardioimmunology (27). Colchicine also has been investigated to attenuate myocardial infarction reperfusion injury and limit the infarct size in mouse models (28). However, clinical studies in patients with STEMI after primary percutaneous intervention showed no differences between groups in the primary outcome of LGE infarct size on CMR (*p* = 0.87), biomarkers of inflammation (including C-reactive protein), or myocardial injury (29). M2-like macrophages, natural killer cells, regulatory T cells, and chimeric antigen receptor (CAR) macrophages are promising targets for therapeutic intervention to reduce fibrosis and infarct size (27, 30).

It is noteworthy that SCAD patients may encounter life-threatening ventricular arrhythmias (presented in 3%–11%) and SCD. Nevertheless, information on the application of implantable cardioverter defibrillator (ICD) therapy in this group is limited (1, 12, 31). SCAD is not harmless but may lead to myocardial infarction and even SCD (32). The ICD therapy in SCAD should be more emphasized and analyzed because patients with SCD are not well represented in the current published reports for SCAD (33). The efficacy and safety of ICD therapy in SCAD remain unclear. Although it might appear to be a viable treatment alternative, the potential advantages and disadvantages have not been thoroughly comprehended (33). The data published from the Massachusetts General Hospital (MGH) SCAD registry showed that SCAD patients with SCD were more likely to be peripartum, be tobacco users, present with STEMI, receive an ICD, and have a higher incidence of repeat SCAD (33). The timing of placement of ICD ranged differently for primary and secondary prevention in inducible ventricular tachycardia (VT) and for the recurrent episodes of non-sustained VT (33). The accordance between ICD implantation and the value of left ventricle ejection fraction (LVEF) in primary prevention is not explained. Data currently support ICD therapy only for secondary prevention in the absence of reversible causes and for primary prevention in patients with persistently low LVEF (31). The reason for the limited data about ICD in SCAD is that lesions usually heal spontaneously, and LVEF is often preserved or recovers shortly after SCAD presentation (31).

In the research of Ciliberti et al. (34), which is a UK nationwide autopsy-based registry of SCD, SCAD victims were mainly women. The majority of SCAD victims were Caucasian and experienced SCD during the peripartum period after the acute onset of symptoms. Interestingly, none of the patients had fibromuscular dysplasia (FMD) in their coronary arteries. In addition, only a quarter of the SCAD patients suffered from myocardial infarction. It is worth noting that pregnancy-associated SCAD can be extensive, affecting the left anterior descending coronary artery and multiple vessels, leading to a significant reduction in LV ejection fraction, which is a reflection of large areas of ischemia and, eventually, myocardial infarction (24).

There is a strong indication that female sex hormones are involved in the marked preponderance of SCAD in fertile women, particularly in the peripartum period. SCAD events related to pregnancy are typically seen in the third trimester or shortly after childbirth. Nonetheless, there have been instances where SCAD has been observed in the first trimester and later in the postpartum period, particularly among breastfeeding women. During the peripartum phase, when female sex hormones are at high levels, estrogen and progesterone receptors in the coronary vasculature may weaken the vascular wall and cause dissection by mediating changes in the connective tissues (32).

## 5. LV function in SCAD patients

CMR was the reference standard for the *in vivo* quantification of LV function between 2 and 9 days after AMI, and revascularization and cine imaging were used to determine LV volumes and global and regional function at baseline and in follow-up (23).

According to Al-Hussaini et al., reducing LVEF and increasing end-diastolic volume (EDV) and end-systolic volume (ESV) of LV were found in SCAD patients compared with matched healthy controls (20). There was no significant difference in infarct size or ESV although female SCAD patients with STEMI had significantly higher LVEF (55.5 ± 7.2% vs. 50.9 ± 10.7%; *p* = 0.0094) and higher EDV (90.2 ± 16.0 ml/m^2^ vs. 82.2 ± 20.6 ml/m^2^; *p* = 0.0260) compared with female Type 1 STEMI patients (20).

When assessing LV function, echocardiography is a commonly used and accessible diagnostic tool (35). In cases of SCAD, most patients have either no myocardial infarction or a small one, and their LVEF is preserved (35). A study by Franco et al. in 2017 found that approximately 26% of SCAD patients with acute symptoms experience a slight decrease in LVEF below 50%, while approximately 5.1% have an LVEF below 40% (36). However, LVEF can be transiently reduced at first evaluation, which is often disproportionately affected compared with the extent and location of SCAD, with subsequent improvement (36).

The Spanish Registry on SCAD (SR-SCAD) (37) discovered differences in clinical characteristics and angiographic findings between SCAD patients with reduced LVEF (<50%) and those with preserved LVEF (≥50%). Patients with SCAD and reduced LVEF <50% presented more often with anterior STEMI and multi-segment involvement coronary disease (37). During the follow-up (median follow-up of 28 months), no significant differences in overall major adverse cardiac and cerebrovascular events (MACCEs) were observed between groups with reduced and preserved LVEF in this registry (37). MACCE was defined as death, heart failure admission, myocardial re-infarction, unplanned coronary revascularization, or stroke. However, SCAD patients with reduced LVEF had higher mortality rates (9% vs. 0.7%, *p* < 0.001) and readmission rates for heart failure (4% vs. 0.3%, *p* = 0.01) (37).

When assessing LV function, wall motion abnormality (WMA) is one more diagnostic parameter in SCAD. Al-Hussaini et al. (20) discovered that SCAD patients have a high percentage (85.6%) of WMA, corresponding to the arterial distribution of SCAD. While most participants experience WMA during the acute stage of SCAD, they also maintain a global value of LVEF (35).

In addition to LVEF and WMA, global longitudinal strain (GLS) and regional strain may be useful diagnostic parameters (38, 39) and prognosis parameters in SCAD survivors (40). The regional longitudinal strain could be impaired in the involved coronary territory and the GLS, which are a more precise prognostic diagnostic measure for patients with preserved LVEF >50% (40, 41). Before cardiac catheterization, an echocardiogram may influence pre-test probability, showing regional WMA or reduced strain in the involved coronary territory. Nevertheless, its role in differential diagnosis and multimodality imaging is quite limited.

## 6. Time for reassessing cardiac function and healing of coronary arteries

According to Hayes et al. (12), reassessing the cardiac function in patients with SCAD within 3 months of their initial diagnosis is recommended. This is important, especially for those with reduced LV function during AMI (14). Regular monitoring and assessment of cardiac function can help determine the progression of the condition and make appropriate treatment decisions.

Routine invasive angiography in asymptomatic patients after SCAD is not recommended (42). However, it may not be necessary in asymptomatic patients unless there are specific indications or concerns.

Nevertheless, for medically managed patients with SCAD involving the proximal-to-mid-coronary vessel dissections, CTA imaging can play a role in confirming healing, particularly for type 1 dissections, or when considering discontinuation of antiplatelet therapies (43). Cardiac CTA is a non-invasive imaging technique that can provide detailed information about the coronary arteries, and it can be used to assess the healing of the dissection and guide treatment decisions.

## 7. Normalization of LV function

More than 50% of SCAD patients have subsequent normalization of myocardial function, WMA, and LVEF in the follow-up assessment (36).

It was hypothesized that SCAD could result in prolonged myocardial ischemia in the territory subtended by the dissected artery, potentially causing myocardial stunning to a greater degree than myocardial infarction. Healing of the vessels leads to improvement of the LV function (36). Possible mechanisms of resolution of myocardial function may be due to reabsorption of intramural hematoma or healing of the dissected coronary artery. However, other possible mechanisms of recovery of the dissected arteries are collateral blood vessel circulation and medical therapy for LV remodeling and improving LV function and WMA (e.g., β-blockers and angiotensin-converting enzyme inhibitors) (36).

The pathophysiological mechanism of occurrence of infarct size is not the same in SCAD STEMI and Type 1 STEMI. Kotecha et al. (44) found that SCAD patients often have completely healed lesions at late follow-up without reperfusion intervention. The revascularization strategy did not improve LVEF in SCAD patients. This may explain why there are differences in myocardial function normalization and infarct size reduction across various clinical entities of SCAD.

**Figure 5:**
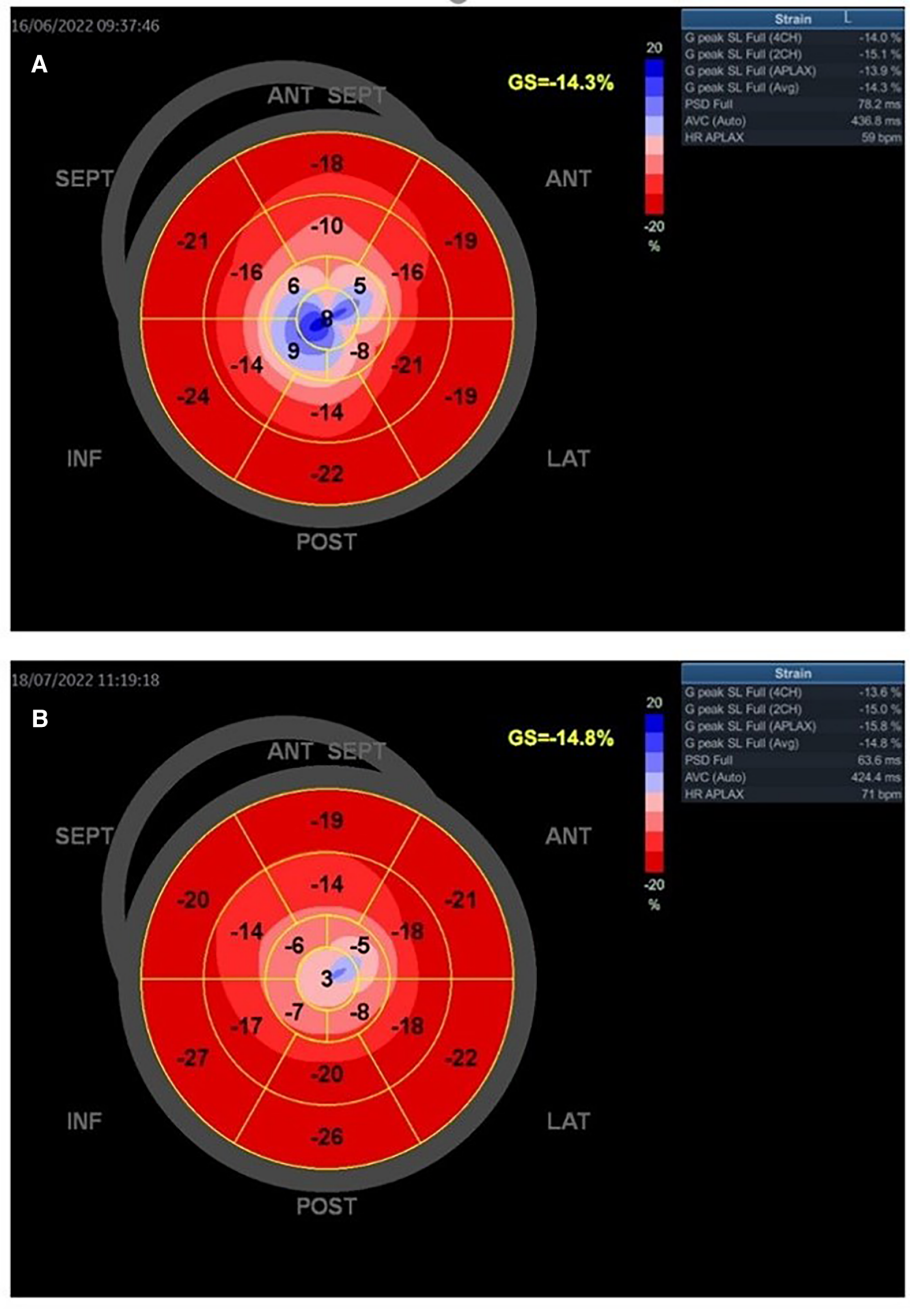
A young woman presented with STEMI anterior localization and SCAD type 4 on the left anterior artery. She underwent treatment with PTCA. The baseline GLS and regional longitudinal strain were measured, and the baseline LVEF was found to be 38% (**A**). GLS and regional longitudinal strain in follow-up after 1 month were presented in one young woman in part (**B**). The control LVEF value was 48%.

**Figure 6:**
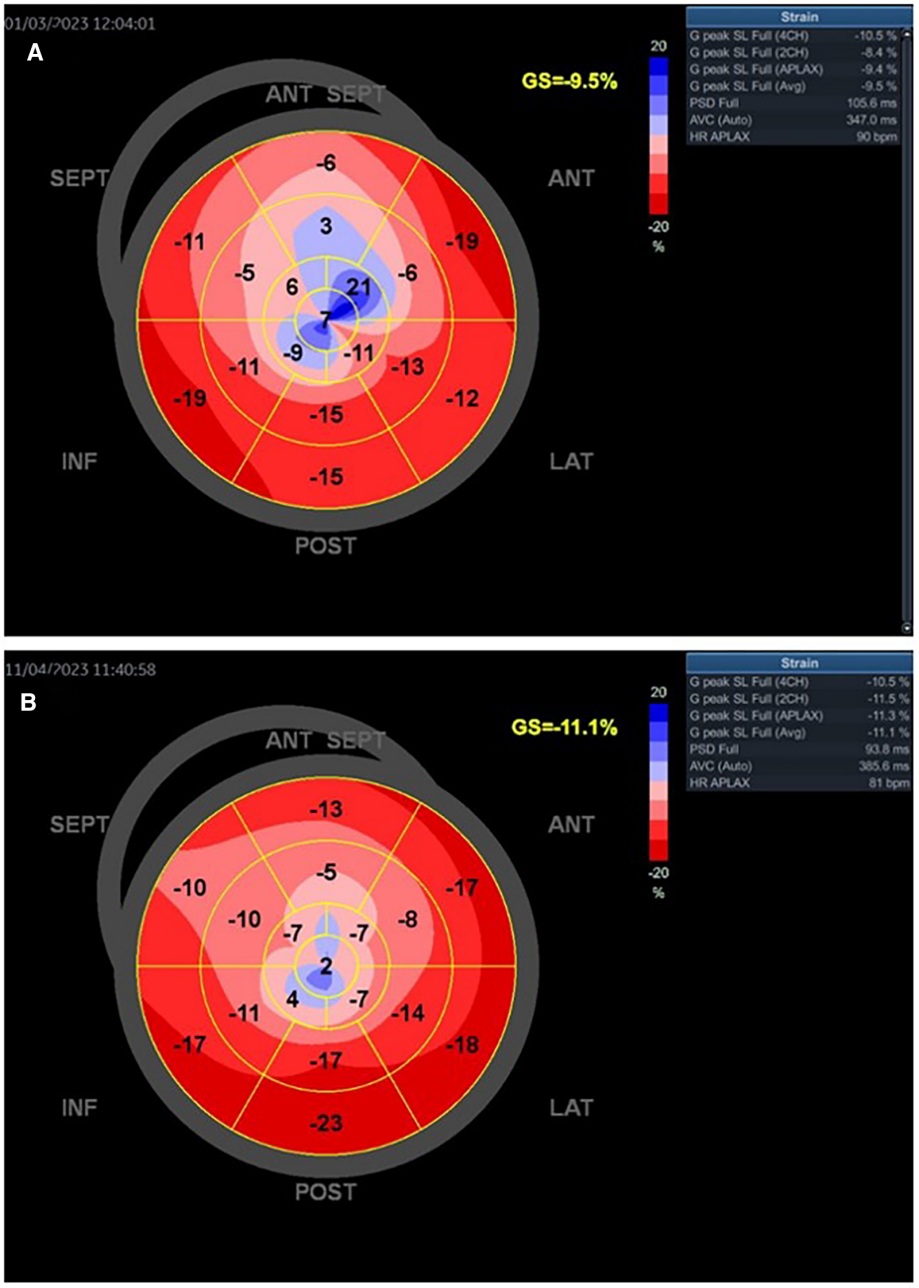
The baseline GLS and regional longitudinal strain in one young woman with SCAD type 3 presented as STEMI anterior localization who was treated with PCI and implanted four stents. The baseline LVEF was 43% (**A**). The improved GLS and regional longitudinal strain were seen in the follow-up of the patient after 1 month (**B**). The control LVEF value was 53%.

**Figure 7:**
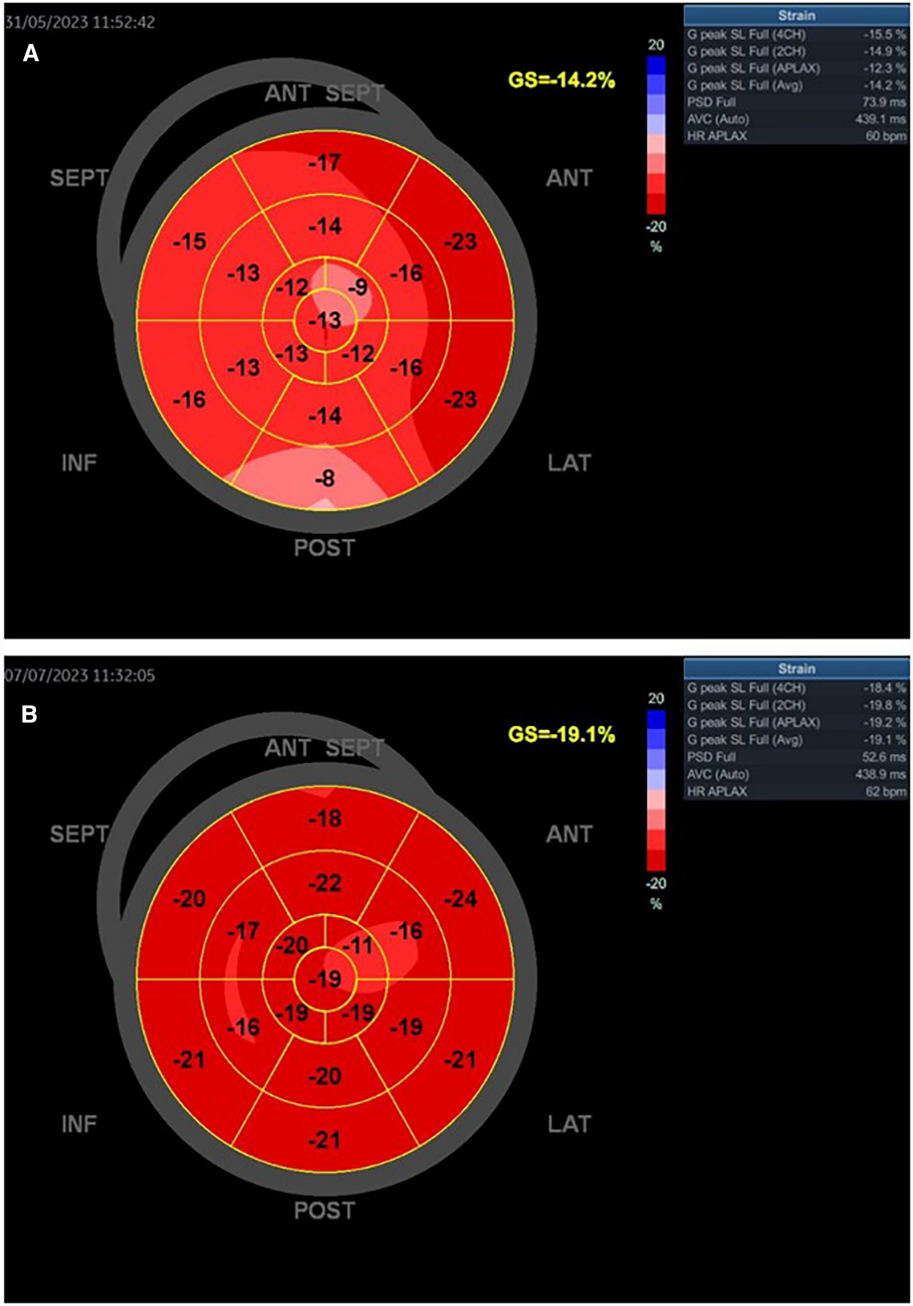
Patient with SCAD type 2A of left anterior artery treated with medical therapy. The baseline LVEF was 52% (**A**). Slightly improved GLS and regional longitudinal strain were seen in the follow-up of the patient after 1 month (**B**). The control LVEF was 65%.

Figures 5–7 show GLS and regional longitudinal strain, providing valuable information on the mechanical deformation of the LV in three SCAD female patients before and after 1 month of follow-up.

Figure 5 presents the GLS and regional longitudinal strain of a woman with SCAD of left anterior artery (LAD) type 4 and reduced LVEF at baseline and follow-up. She was treated with percutaneous transluminal coronary angioplasty (PTCA), and the figures depict the changes in her condition from baseline to the 1-month follow-up.

Figures 6A,B present the values of LVEF and GLS of a woman with SCAD of LAD type 3. She had mildly reduced LVEF on baseline (LVEF 43%), was treated with PCI, and had improved GLS and regional longitudinal strain after the 1-month follow-up. The LVEFs, GLS, and regional longitudinal strain are improved in the follow-up of these patients.

On the other hand, Figures 7A,B present the values of a woman with SCAD LAD type 2A treated only with medical therapy. She had preserved LVEF and slightly reduced GLS, without myocardial scar at baseline and after the 1-month follow-up. These parameters are also improved after the 1-month follow-up.

Based on the previous examples of various types and management of SCAD patients, it seems that the process of normalizing LV function could be complex and require further investigation.

Nevertheless, using advanced diagnostic echocardiography techniques, such as mechanical deformation and CMR assessment, can potentially provide significant benefits in monitoring SCAD patients.

## 8. Limitations

Despite our attempts to provide a comprehensive, up-to-date summary of the relevant aspects of patient monitoring and follow-up after a SCAD event and the role of imaging modalities, we must acknowledge that the lack of prospective outcome trials or large-scale observational studies that could provide solid evidence for the clinical practice largely limits the scope of the current knowledge. Therefore, most of the current knowledge is based on smaller observational studies without a long-term follow-up. Furthermore, despite our diligence in summarizing available data, some of the pertinent research may have been unintentionally missed. These limitations call for future high-volume prospective and randomized studies of following patients with SCAD to provide high-quality evidence to inform clinical practice.

## 9. Conclusion

Myocardial injury and LV function after SCAD can be variable parameters and are sometimes essential in the follow-up of patients with SCAD. Advanced echocardiographic and CMR techniques may provide an excellent opportunity to monitor myocardial scarring and LV function and help guide the management of patients with SCAD. Finding accurate prognostic diagnostic parameters for the occurrence of heart failure, malignant arrhythmias, and SCD in patients with SCAD may be of crucial importance and should be the subject of future research.
